# Factor structure and measurement invariance of the Depression anxiety stress scale (DASS-21) in Chinese left-behind and non-left-behind children: an exploratory structural equation modeling approach

**DOI:** 10.1186/s12889-024-19160-y

**Published:** 2024-06-21

**Authors:** Wei Chen, Kaijing Peng, Meihui Gao, Zhu Meng, Luolan Wang, Yaxi Liao

**Affiliations:** 1 School of Psychology, Guizhou Normal University, Guiyang, China; 2 Center for Big Data Research in Psychology, Guizhou Normal University, Guiyang, China

**Keywords:** DASS-21, Chinese left-behind children, ESEM, Measurement invariance, Longitudinal invariance

## Abstract

**Background:**

Comprehensive data has shown that adolescents often suffer from depression, anxiety, and low self-esteem, and are in a particularly fragile stage of psychological, physiological, and social development. Left-behind children in particular tend to have significantly higher, state anxiety and depression compared to non-left-behind children. The Depression, Anxiety, and Stress Scale (DASS-21) is an effective tool for evaluating depression, anxiety, and stress, and is used to measure levels of depression, anxiety, and stress in groups from a variety of backgrounds. The purpose of this study was to determine the effectiveness, reliability, and measurement invariance of the DASS-21 in Chinese left-behind children.

**Method:**

The test and re-test method was used (*N* = 676), and the exploratory structural equation model (Mplus v.8.3) used to verify basic measurement models. For measurement invariance, the configural, weak, strong, and strict models were tested. The reliability of the DASS-21 was also tested using the collected data.

**Results:**

Analysis results showed that the DASS-21 had a stable three-factor structure in the sample of left-behind children in China. The measurement invariance test showed that gender and time not only had strong invariance, but also strict invariance. The results of cross left and non-left invariance indicated a lack of strict invariance. Finally, the McDonald’s omega coefficient of the DASS-21 total scale was 0.864, and the internal consistency of each subscale was also good.

**Conclusions:**

The DASS-21 is shown to be an effective and reliable tool for measuring depression, anxiety and stress in Chinese left-behind children.

## Introduction

Of all mental disorders, emotional disorders have the highest prevalence worldwide [1], and the experience of depression, anxiety and stress tend to exacerbate the risk of developing emotional disorders [2]. Depression manifests as having low interest in daily activities, which is usually defined as experiencing a low level of enthusiasm, despair, inferiority complex and low motivation [3]. Anxiety is a subjective and uncomfortable feeling of stress or worry, distinct from fear or excitement caused by the expectation of danger [4] and uncontrolled anxiety will aggravate feelings of depression [5]. Stress is a negative emotional experience which is accompanied by predictable physiological, cognitive and behavioral changes which are adopted with the aim of changing or resolving stress events or to adapt to their effects [6]. The stress process model shows that vulnerability factors such as depression or anxiety can affect exposure or response to stress [7]. Stress shows a high correlation with both depression and anxiety, and has been proved to be a risk factor for the development of both, which will have a significant negative impact on one’s quality of life and economic costs [8]. Evidence shows that these mental health problems are directly related to social and physical problems such as family relationship disorders, higher rates of suicide, poor grades and use of illegal drugs [9–11], and it is no surprise that economically disadvantaged developing countries have reported the highest prevalence of depression and anxiety symptoms [12]. Moreover, comprehensive data has shown that depression, anxiety and low self-esteem are all particularly prevalent among adolescents [13].

Adolescents are at a particularly vulnerable stage in terms of their psychological, physical, and social development. Some countries have reported particularly high rates of self-harm and deteriorating mental health among adolescents [14–16], with adolescents being more prone to depression and suicidal behavior. Within the demographic of adolescents, left-behind children are even more emotionally unstable and at higher risk of mood disorders. As a particularly vulnerable group created by the outcomes of China’s rapid urbanization and economic development, the physical and mental health of this group of youth has long been of great concern [17]. Left-behind children are youth under the age of 18 who have been raised, educated, and managed by other family members while their parents migrate to urban areas to increase their economic opportunities [18, 19]. Parental labor migration in China tends to undermine family integrity, depriving left-behind children of normal parental resources, attachments, and family interactions. Studies have shown that parental migration has a negative impact on children’s mental health, leading to a high prevalence of low self-esteem, loneliness, anxiety, depression, and even suicidal ideation [20]. Compared to their peers in the general population, lack of traditional family upbringing, limited parental care, and reduced parent-child contact [21] have all emerged as important factors contributing to the mental health challenges experienced by left-behind children in rural China, and depression has become one of the most common psychological problems among left-behind adolescents [22]. Vulnerable children are more likely to develop depression than normal children and several studies have reported higher prevalence rates of depression or anxiety among left-behind children than among other children [23, 24], and have suggested that the separation from their parents increases their vulnerability to mental health problems. Existing research has shown that depression is an elevated emotional problem among left-behind children [25]. Cheng et al. [26] systematically reviewed the prevalence and predictors of depression and anxiety among left-behind children and found that left-behind children had higher rates of psychological depression/anxiety compared to their peers, with prevalence rates ranging from 12.1 to 51.4% for depression and 13.2–57.6% for anxiety. In addition to internalized problems such as depression, left-behind children also show externalized problems due to their special situation and status. Leading to even greater negative impacts on their psychological adjustment [27]. In fact, the results of one study on left-behind children showed that, stressful life events significantly predicted left-behind children’s internalized and externalized problematic behaviors, such as such loneliness [28].

It has been reported that there are more than 68 million left-behind children in China [29], and a 2014(year) meta-analysis reported a depression rate in left-behind children of 26.4% [30]. Compared with those living in intact family environments, children who have been separated from their parents for long periods of time are more likely to suffer from a variety of problems, including low self-esteem, emotional trauma, depression, and anxiety [31], as economic hardship and social discrimination are additional sources of stress that may also exacerbate the risk of depression among left-behind children. Overall, these results suggest that left-behind children face an increased risk of mental health problems, highlighting an urgent need to develop better preventive measures and more effective emotional management strategies for this demographic. For this reason, it is important to be able to effectively assess negative emotions such as depression, anxiety, and stress in Chinese left-behind children.

Clark and Watson proposed a tripartite model of anxiety and depression comprising general distress, physiological hyperarousal (specific anxiety), and anhedonia (specific depression), to differentiate between common mood disorders such as depression, anxiety, and stress [32]. Using this as a basis, Lovibond and Lovibond, compiled the Depression Anxiety Stress Scale (DASS) [33]. The DASS is a self-reporting instrument designed to maximize the difference between depression and anxiety symptoms while also revealing their common characteristics, namely stress. The DASS is composed of three subscales, depression (assessment of anxiety emotional state), anxiety (assessment of arousal state), and stress (assessment of negative emotions towards stressors and general tension), which are measure using a total of 42 items. The screening tool has been widely used in both non-clinical [34] and clinical groups [35]. The short version of the DASS (DASS-21), consisting of 21 items, has been proven to have even better psychometric characteristics than the original scale, and has shown good internal consistency and reliability (Cronbach’s α = 0.74 to 0.93) [36, 37], showing more consistency and uniqueness in assessing depression, anxiety, and stress. Numerous studies have also tested the structural effectiveness of the DASS-21, with most proving the stability of the three-factor structure of the DASS-21 in different groups. Several other short versions of the DASS have also since been created (see Table 1), and it has been translated into 45 different languages (e.g., Bangla, Chinese, Arabic, etc.) [38]. The 21-item version of the DASS is the most-used in clinical and non-clinical group studies around the world due to its good psychometric parameters [8, 39], and its effectiveness in assessing depression, anxiety, and stress in both adolescents and adults [40] across different social and cultural backgrounds and sample groups [41]. In the Chinese context, the DASS-21 has been proved to be reliable [42, 43], but its application in the left-behind children demographic has been limited.

**Table 1:** Shortened versions of the DASS-21

DASS versions	Author	Years	Factor Structure	Sample	Age range (M, SD)	Reliability	Item removed
DASS-20	Park et al.	2020	Three factor	New South Wales, Australia ASD (*N* = 123)	16-46Years (M = 23.38, SD = 6.95)	-	item2
DASS-20	Medvedev et al.	2018	Three factor	New Zealand university students and general population (n1 = 200, n2 = 200)	17-95Years (M = 38.09, SD = 20.40)	-	item5
DASS-20	Amin et al.	2014	Three factor	Klang Valley Female nurse (*N* = 453)	25-55Years (M = 30.62, SD = 5.56)	α-D = 0.76, α-A = 0.77, α-S = 0.81, α-T = 0.90	item3
DASS-19	Leung et al.	2009	One factor	Chinese college students (*N* = 206)	-	Person reliability = 0.92	-
DASS-18	Lan et al.	2020	Three factor	Vietnam students (*N* = 2000)	-	α-D = 0.882, α-A = 0.807, α-S = 0.842, α-T = 0.909	item2, item4, item7
DASS-18	Oei et al.	2013	Three factor	Asian employees (*N* = 2300)	(M = 30.46)	α-D = 0.86, α-A = 0.81, α-S = 0.70, α-T = 0.91	item9, item10, item11
DASS-17	Ali and Green	2019	One factor	Egyptian inpatient drug users (*N* = 149)	19-60Years (M = 32.5, SD = 6.8)	α-T = 0.881	item2, item6, item14, item18
DASS-14	Wise et al.	2017	Three factor	Melbourne rehabilitation health professionals (*N* = 343)	18-74Years	α-D = 0.88, α-A = 0.73, α-S = 0.84	item2, item5, item8, item9, item13, item15, item20
DASS-13	Murad	2022	Three factor	Jordan adults (*N* = 935)	18–72 Years (M = 33.55, SD = 13.43)	-	item3, item4, item5, item8, item12, item14, item15, item19
DASS-12	Osman et al.	2014	Three factor	Malaysia secondary school student (*N* = 2850)	13-17Years	α-D = 0.68, α-A = 0.53, α-S = 0.52, α-T = 0.76	item5, item8, item9, item11, item12, item13, item15, item16, item20
DASS-10	Halford et al.	2021	Three factor	Australia Brisbane clinical and community sample (n1 = 2120, n2 = 376)	n1: M = 35.6, SD = 14.3 n2: M = 32.8, SD = 10.3	α-T = 0.89	item5, item6, item8, item9, item14, item15, item16, item20, item21
DASS-9	Yusoff	2013	Three factor	Malaysia medical students (*N* = 706)	18-21Years (M = 19.17, SD = 0.70)	α-D = 0.723, α-A = 0.52 α-S = 0.573, α-T = 0.550	item1, item2, item3, item4, item8, item12, item13, item14, item17, item18, item19, item20
DASS-8	Ali et al.	2021	Three factor	Saudi Arabia psychiatric patients and psychiatric patients (n1 = 168, n2 = 992)	≥ 18Years	α-T = 0.94	item1, item2, item3, item4, item5, item6, item7, item11, item14, item17, item18, item19, item21

Note: α-D = reliability of depression; α-A = reliability of anxiety; α-S = reliability of stress; α-T = reliability of total scale

One study validating the DASS-21 found that the measure showed gender sensitivity, with women scored higher on the total measure and on its subscales [44], although Jiang et al. [45] examined the gender measurement invariance of the DASS-21 among 1,532 Chinese hospital staff (74.4% female; mean = 31.97 years, (*SD* = 9.70), but it is not yet clear whether this also applies to Chinese children. In fact, the mental health status of left-behind children is in a dynamic process of change as they grow up. As such, research on clinical interventions and their effects on negative emotions should be compared and tested using longitudinal results [43], making it even more important to be able to reliably evaluate depression, anxiety, and stress across time, using an instrument has acceptable longitudinal invariance.

Confirmatory factor analysis (CFA) has been widely used to study the measurement characteristics of the DASS-21 [46], but some researchers believe that CFA has limitations in its ability to test the multi-factor measurement model [47], particularly compared to exploratory structural equation modeling(ESEM). ESEM combines the functions of exploratory factor analysis (EFA) and CFA [48], and can simultaneously estimate the load of the main factors of subjects as well as the load of some other factors, even if they are relatively small. When verifying the factor model, ESEM is also easier to implement than CFA, and tends to obtain results closer to the real situation, allowing for more advanced statistical analysis. For all these reasons, when validating the DASS-21 in Chinese left-behind children, ESEM may be more suitable than CFA, especially with the advantages of ESEM in testing measurement invariance.

Thus, the current study aimed to examine the measurement characteristics of the Chinese version of the DASS-21 in Chinese left-behind children. In addition to the traditional CFA method, the ESEM method was also used to analyze the structural validity of the DASS-21. At the same time, cross-gender, cross-left-behind equivalence, and longitudinal measurement invariance were also studied within the ESEM framework. Finally, the internal consistency and reliability of the DASS-21 were explored.

## Method

### Participants

Participants in the current study were students from Guizhou Province, China. They were invited to participate voluntarily, and each participant was invited to voluntarily provide their student ID number and name when completing the questionnaire for the first time if they were interested in participating in the data collection at the second time point, six months later. After excluding children who submitted invalid answers, a total of 676 children aged 10 to 16 years participated in the study (mean age = 12.58 years; *SD* = 0.94; 52.70% boys; 42.90% left-behind children). After an interval of six months, the student participants completed the follow up questionnaires.

### Procedure

As the participants in this study were under the age of 18, the school first informed the students and their parents of the purpose and process of the survey one week in advance, and the data collection was carried out in the students’classroom at school, and under the supervision of the participants’teacher. The students were assured that their answers would be kept strictly confidential and that their choice to participate in theresearch would not affect their academic performance. After obtaining the informed consent of both the students and their guardians, the investigation was carried out during a 15-minute session held after class. The teacher in charge of the survey read out the instructions and explained the measurement purpose in a controlled manner to reduce the risks of guess-related deviations. The teachers explained any questions that the students did not understand to reduce or avoid measurement errors caused by students’understanding bias. After the students had completed the questionnaires, they were collected by the teacher and handed onto the survey leader, who sent them back to the research team. The researchers checked the questionnaires carefully and excluded any incomplete or abnormal answers.

This study conforms to the ethical standards of the 2013 Helsinki Declaration and was approved by the Committee of the School of Psychology of Guizhou Normal University (ID: 20,201,018), and informed consent was obtained from all participants.

### Measures

The study used the Chinese version of the DASS-21 scale, first introduced by Chinese scholar Gong et al. [42]. The Depression and Anxiety Stress Scale-21 (DASS-21; Lovibond [33]; Chinese version developed by Gong et al. [42]) is a 21-item self-reported questionnaire, used to evaluate the symptoms of depression (e.g., “I felt that I have nothing to look forward to”), anxiety (e.g., “I was worried about situations in which I might panic and make a fool of myself”), and stress (e.g., “I tended to over-react to situations”), with seven items used to measure each dimension. Participants are asked to score each item using a Likert scale ranging from 1 (“Did not apply to me at all”) to 4 (“Applied to me very much or most of the time”) regarding the applicability of each item to their experience of the past week. The total of each subscale is calculated by adding the scores of all seven items and multiplying the sum by two. The total measurement score can range from 0 to 42, and the higher the score, the stronger the respondent’s negative emotional experience.

### Statistical analysis

EpiData3.1 was used to encode the questionnaire data manually, after which it was converted to dta format. The demographic characteristics and DASS-21 scores were analyzed using SPSS v26 software. The distribution of data was analyzed by calculating the skewness and kurtosis levels, following the recommendations that, when the absolute value of kurtosis is less than 10 and the absolute value of skewness is less than 3, the data can be accepted as normal distribution.

Mplus v8.3 software was used to perform the CFA, ESEM and measurement invariance. In terms of data distribution, the results showed that the data was subject to normal distribution. Therefore, the maximum likelihood estimation method was adopted. ESEM was then conducted to verify the structure of the DASS-21. Using geomin oblique rotation, parameter estimation used the maximum likelihood method. The CFA results were also compared with the ESEM results. The Chi-square test is too sensitive to evaluatemeasurement invariance in large samples (*N* > 300) [49], as even small differences might produce significant results with the increase of sample size, so it was not considered to be useful as the main indicator of model fitting [50]. However, a value of χ^2^/*df* of less than 5 was considered to be acceptable. The following indicators were used to test for model fit: comparative fit index (CFI), Tucker-Lewis index (TLI), standardized root mean square residual (SRMR), root mean square error of approximation (RMSEA), with CFI > 0.90, TLI > 0.90, SRMR close to or less than 0.08, and RMSEA < 0.08 indicating an acceptable model fit [51]. Next, ESEM was used to test the effects of gender, left-behind status, and time invariance on the DASS-21. Four models were established, namely, configural invariance, weak, strong and strict equivalence, and the changes between the models were compared. The evaluation criteria of measurement invariance were as follows: $\Delta$CFI < 0.010, $\Delta$TLI < 0.010 and $\Delta$RMSEA < 0.015 [52]. If at least two of the three changes of the fitting index in the nested model met the cut-off criteria, the scale was believed to meet the requirements for measurement invariance [53].

Finally, to test the internal consistency of the DASS-21, the McDonald’s omega value was calculated using Jamovi v2.3.16 software. According to Revelle and Zinbarg [54], the McDonald’s omega provides a more accurate approximation of the reliability of a scale, with the minimum required McDonald’s omega value being more than 0.60 [55].

## Results

### Descriptive statistics

The data collected at the first time point was used to conduct descriptive statistical analysis of the DASS-21. The mean of each item was between 1.39 and 2.59, and the *SD* was between 0.75 and 1.00. The average total score of the scale was 41.65, and the standard deviation was 18.80. The absolute value of the skewness coefficient was between 0.01 and 2.03, while the absolute value of the kurtosis coefficient was between 0.08 and 3.27.

### DASS-21 factor structure

The applicability of CFA and ESEM were compared for the DASS-21 and the necessity of using ESEM was verified. As seen in Table 2 in the traditional CFA model fitting index, TLI and CFI did not meet the fitting standard, indicating that the model fitting data was poor. However, the ESEM model fitting index reached the ideal level (χ^2^/*df* = 1.896, TLI = 0.932, CFI = 0.952, SRMR = 0.029, RMSEA = 0.036), for both boys(χ^2^/*df* = 1.385, TLI = 0.943, CFI = 0.960, SRMR = 0.034, RMSEA = 0.033) and girls(χ^2^/*df* = 1.481, TLI = 0.927, CFI = 0.948, SRMR = 0.039, RMSEA = 0.039) also reaching the standard, indicating that the data and model fit well. In other words, a stable three-factor structure was established for the different groups. Therefore, the next measurement invariance test was carried out using the ESEM framework.

**Table 2:** Comparison of CFA and ESEM Fitting Indexes (*N* = 676)

Model	χ^2^	df	χ^2^/df	TLI	CFI	SRMR	RMSEA(90%CI)
CFA	477.664	186	2.568	0.882	0.895	0.042	0.048(0.043, 0.054)
ESEM	284.436	150	1.896	0.932	0.952	0.029	0.036(0.030, 0.043)
ESEM (male)	207.705	150	1.385	0.943	0.960	0.034	0.033(0.021, 0.043)
ESEM (female)	222.199	150	1.481	0.927	0.948	0.039	0.039(0.027, 0.049)

*Notes*: χ^2^ = chi-square goodness of fit; *df* = degrees of freedom; TLI = Tucker–Lewis index; CFI = comparative fit index; SRMR = standardized root mean square residual; RMSEA = root mean square error of approximation; 90% CI = 90% confidence interval for RMSEA

### Measurement invariance across genders

In the cross-gender test, the model fitting statistics of configural invariance were satisfactory (TLI = 0.936, CFI = 0.954, SRMR = 0.036, RMSEA = 0.036), which supported the baseline model of boys and girls. With the establishment of configural invariance, the weak equivalence test was carried out. The fitting results of the weak equivalence test showed that $\Delta$TLI, $\Delta$CFI and $\Delta$RMSEA were 0.007, 0.014 and 0.002 respectively(see full results in Table 3). Although $\Delta$CFI = 0.014 > 0.01, considering the results of the other two indicators, the weak equivalence model of gender was also deemed to be acceptable. The fitting results of strong and strict equivalence tests showed that $\Delta$TLI, $\Delta$CFI and $\Delta$RMSEA were all < 0.01, which supported gender equivalence.

**Table 3:** Multi-Group ESEM Comparison Nested Model Fitting Index (Gender Invariance, *N* = 676)

Model	χ^2^	df	χ^2^/df	TLI	CFI	SRMR	RMSEA (90% CI)	$\Delta$TLI	$\Delta$CFI	$\Delta$RMSEA
Configural invariance	429.905	300	1.433	0.936	0.954	0.036	0.036(0.028–0.043)	-	-	-
Weak invariance	523.726	354	1.479	0.929	0.940	0.046	0.038(0.031–0.044)	0.007	0.014	0.002
Strong invariance	554.701	372	1.491	0.927	0.935	0.047	0.038(0.031–0.045)	0.002	0.005	0.000
Strict invariance	584.842	393	1.488	0.927	0.932	0.051	0.038(0.031–0.044)	0.000	0.003	0.000

*Notes*: χ^2^ = chi-square goodness of fit; *df* = degrees of freedom; TLI = Tucker–Lewis index; CFI = comparative fit index; SRMR = standardized root mean square residual; RMSEA = root mean square error of approximation; 90% CI = 90% confidence interval for RMSEA; $\Delta$TLI = change in TLI; $\Delta$CFI = change in CFI; $\Delta$RMSEA = change in RMSEA

### Measurement invariance across left-behind and non-left-behind

Table 4 shows the configural invariance ESEM results of the potential variables between left-behind and non-left-behind children. The baseline model was established according to the fitting index (TLI = 0.917, CFI = 0.941, SRMR = 0.037, RMSEA = 0.041). A small difference was found in the change of the fitting index between the configural invariance and the weak equivalence model($\Delta$TLI = 0.006, $\Delta$CFI = 0.006, $\Delta$RMSEA = 0.002), thus the weak equivalence model was established. In the strong and strict equivalence models, only some indexes change within the range of < 0.01, and some fitting indexes did not meet the cut-off standard.

**Table 4:** Multi-Group ESEM Comparison Nested Model Fitting Index (Left-Behind and Non-Left-Behind Groups Invariance, *N* = 676)

Model	χ^2^	df	χ^2^/df	TLI	CFI	SRMR	RMSEA (90% CI)	$\Delta$TLI	$\Delta$CFI	$\Delta$RMSEA
Configural invariance	466.743	300	1.556	0.917	0.941	0.037	0.041(0.033–0.048)	-	-	-
Weak invariance	537.171	354	1.517	0.923	0.935	0.044	0.039(0.032–0.046)	0.006	0.006	0.002
Strong invariance	536.611	372	1.443	0.777	0.802	0.089	0.074(0.060–0.088)	0.146	0.133	0.035
Strict invariance	551.290	393	1.403	0.797	0.810	0.089	0.071(0.056–0.084)	0.020	0.008	0.003

*Notes*: χ^2^ = chi-square goodness of fit; *df* = degrees of freedom; TLI = Tucker–Lewis index; CFI = comparative fit index; SRMR = standardized root mean square residual; RMSEA = root mean square error of approximation; 90% CI = 90% confidence interval for RMSEA; $\Delta$TLI = change in TLI; $\Delta$CFI = change in CFI; $\Delta$RMSEA = change in RMSEA

### Longitudinal measurement Invariance

Using time as a group index (i.e., first time point = 1, second time point = 2), the longitudinal measurement invariance of the DASS-21 was tested in a sample of 290 left-behind children. The configural (TLI = 0.931, CFI = 0.943, SRMR = 0.050, RMSEA = 0.038), weak ($\Delta$TLI = 0.002, $\Delta$CFI = 0.002, $\Delta$RMSEA = 0.001), strong ($\Delta$TLI = 0.001, $\Delta$CFI = 0.001, $\Delta$RMSEA = 0) and strict equivalence models($\Delta$TLI = 0.005, $\Delta$CFI = 0.001, $\Delta$RMSEA = 0.001) were very satisfactory, which supported the longitudinal invariance of the DASS-21 (see Table 5).

**Table 5:** Multi-Group ESEM Comparison Results of Nested Model Fitting Index (Longitudinal Invariance, *N* = 290)

Model	χ^2^	df	χ^2^/df	TLI	CFI	SRMR	RMSEA (90% CI)	$\Delta$TLI	$\Delta$CFI	$\Delta$RMSEA
Configural invariance	1006.573	711	1.416	0.931	0.943	0.050	0.038(0.032–0.043)	-	-	-
Weak invariance	1072.068	765	1.401	0.933	0.941	0.049	0.037(0.032–0.042)	0.002	0.002	0.001
Strong invariance	1092.338	783	1.395	0.934	0.940	0.049	0.037(0.032–0.042)	0.001	0.001	0.000
Strict invariance	1068.911	750	1.425	0.929	0.939	0.048	0.038(0.033–0.043)	0.005	0.001	0.001

*Notes*: χ^2^ = chi-square goodness of fit; *df* = degrees of freedom; TLI = Tucker–Lewis index; CFI = comparative fit index; SRMR = standardized root mean square residual; RMSEA = root mean square error of approximation; 90% CI = 90% confidence interval for RMSEA; $\Delta$TLI = change in TLI; $\Delta$CFI = change in CFI; $\Delta$RMSEA = change in RMSEA

### Reliability

In terms of internal consistency, the McDonald’s omega coefficient of the DASS-21 total table was 0.864. The McDonald’s omega coefficients of factor 1 (depression), factor 2 (anxiety) and factor 3 (stress) were 0.709, 0.707 and 0.669, respectively.

## Discussion

Many studies have shown ESEM, rather than CFA, may often be more applicable when model testing for data fit [56]. Some researchers have argued that the ESEM model is better able to describe the data, and the results of the current study lend further support to this claim. This study examined the validity and reliability of the Chinese version of the DASS-21 in Chinese left-behind children. Previous studies have found that ESEM is able to overcome the problem of “too strict fitting criteria” other traditional methods and can organically integrate the functions of EFA and CFA [57]. The results of this study show that the ESEM model does indeed have more advantages than the CFA model, achieving a higher fitting index for the current data. These results show that ESEM does act as a more flexible and reliable tool when analyzing scale structures.

In previous studies, the three-factor structure of the DASS-21 has been obtained through CFA [32, 33], however, the CFA validated model fit indicators in this study were poor, which could be due to the presence of many cross-loaded factor items in the Chinese DASS-21. For the factor structure of DASS-21, we first used CFA to test the three-factor model. The results showed that the CFA model did not reach an acceptable level. The correlation between individual factors in CFA was too high, however this result may be due to the aforementioned limitations of the CFA method. Therefore, the ESEM model was used to further test the three-factor model of the DASS-21, and found that the ESEM model did indeed fit well. As in previous studies, a stable three-factor structure was confirmed in this study [39, 58, 59]. According to Marsh et al. [60], CFA should be more minimalist if the model fit indices of CFA and ESEM are similar. Conversely, if ESEM is superior to CFA, then providing no crossload is in fact an overly restrictive condition. As CFA models can be nested within ESEM, the comparisons in the curret study are statistically significant. This method is considered to be one of the most effective ways currently to address the limitations of CFA, allowing for the presence of cross-factors within the multi-factor model, resulting in a more realistic hypothetical multi-factor measurement model. This presents the relationship between the items and factors more realistically, while also accurately presenting the relationships between factors.

This study also preliminarily verified the cross-gender and cross-left-behind equivalence as well as the longitudinal measurement invariance of the DASS-21. After checking the measurement invariance of the scale, comparisons could be made of the different group scores. The gender invariance was tested according to the order of configural invariance, weak, strong and strict equivalence. The baseline model showed that boys and girls shared the same structure, that is, each group had the same factor pattern. Following the establishment of the configural invariance model (i.e., the baseline model), a weak invariance model was established with an equal load of limiting factors. The results support the potential traits and observation indicators of the 21 items in the DASS-21, which all have the same significance between boy and girl populations. The strong invariance model supported the fact that the intercepts of each observation variable in the DASS-21 were the same across the different groups, that is, they had the same reference points between the observation variables of the different groups. Finally, the small changes in fitting indicators between models fully supported the establishment of a strict equivalence model. Millsap [61] has suggested that configural invariance, weak equivalence and strong equivalence models are all valid, which can explain the significance of the cross-group comparisons of the DASS-21. Therefore, our results show that the 21 items of the DASS-21 have the same meaning when measuring the level of depression, anxiety, and stress of either boys or girls, and that the DASS-21 scale has complete strict gender equivalence. This means that the scale evaluates the same structure in either of the two samples, and is effective for use in either gender, and does not require separate standard gender-based scores. These results are similar to Kyriazos et al. [62]. This shows that the gender invariance of the DASS-21 is consistent across different cultural backgrounds. This is in line with the findings of other studies which have proven the gender equivalence of the DASS-21 across different sample groups, such as Lu et al. [63] who demonstrated the complete strong equivalence of the DASS-21 in a sample of Chinese college students, or Gomez et al. [34] who also demonstrated strong equivalence between Australian men and women.

The current study also examined the measurement invariance between the left-behind group and the non-left-behind group, but the results only supported the two models of configural invariance and weak equivalence, with the fitting index between the strong equivalence model and the strict equivalence model changing significantly (> 0.01). Therefore, the DASS-21 was not shown to have cross-left-behind equivalence. This could be due to the correlation between two observational variables being greater than or equal to 1, or due to a linear correlation between more than two observation variables [64]. Given the unique negative emotions experienced by left-behind children as compared to non-left-behind children, the reason for this result may also be that the scale is simply more suitable for the left-behind children group.

The study also tested the LMI of the 21 items in the DASS-21. The results showed that the factor model of the DASS-21 remained constant at both measurement time points over a six-month interval. The results of multiple CFA of the ESEM results showed that the psychometric characteristics of the scale were not affected by time. The above four invariants are valid, indicating that the Chinese version of the DASS-21 has longitudinal measurement invariance across time in the left-behind children group in China. Furthermore, the observation scores of the DASS-21 can be reasonably compared at different time points. To our knowledge, there is currently a lack of research testing the longitudinal invariance of the DASS-21, particularly in the context of left-behind children in China. As such, the results of this study provide further strong evidence for LMI of the DASS-21. To better determine the changes in elastic characteristics such as depression, anxiety, or stress over time, the ability to measure the impacts of timely interventions focused on improving the mental health development of left-behind children would be beneficial.

Finally, the internal consistency and reliability analysis results indicated that the McDonald’s omega values of the DASS-21 total scale and each subscale reached an expected acceptable level. These results are consistent with those of other studies which have also shown that the DASS-21 has good internal consistency reliability [3, 65–70].

In summary, the ESEM method was adopted to evaluate the measurement characteristics of the DASS-21 specifically in left-behind children in China, and the results further expand the practical applications of this measurement tool. Specifically, the DASS-21 was shown to measure negative emotions such as depression, anxiety and stress specifically within the demographic of left-behind children, allowing for more timely interventions to be conducted following the results of the DASS-21, helping practitioners and healthcare providers to better prevent further serious negative emotions for these vulnerable children, and lead to improved development of the mental health of left-behind children.

### Limitations and future directions

The DASS-21 is shown to have good reliability and validity in a sample of Chinese left-behind children, and can be used to assess their levels of depression, anxiety and stress. However, this study still has some shortcomings which should be noted. In terms of analytical methods, ESEM is a structural equation modeling (SEM) method. Similar to the EFA measurement model, SEM contains a similar structure, with the difference being in its measurement modeling, which allows for simultaneous estimation of loadings of a same indicator on multiple factors. However, this does not mean that ESEM can be used to replace any of the other methods. Second, because the ESEM set must be rotated in its entirety when any factor is rotated, all of the topics in an ESEM set can only be set as a whole in relation to the out-of-set variables, which reduces the flexibility of the analysis to some extent. Therefore, the application of ESEM analysis methods must be analyzed on a case-by-case basis.

In terms of samples, first, our longitudinal tracking samples were limited to only a few schools in Guizhou province. Therefore, the sample range is narrow and not large enough, which can affect the general applicability of the results, as a small number of samples cannot represent all potential sample demographics in China. Future studies should expand the sample size to further analyze left-behind children in China using the DASS-21 to confirm and extend the results of the current study. Second, because the current study used only two time points, the number of follow-up surveys was not sufficient. The mental health status of left-behind children is in a dynamic process of change, making it difficult to determine whether their depression, anxiety, and stress status will change over a longer period of time. Future studies should adopt a longer time interval between measurements to better determine the changes of elastic characteristics over time. Finally, although the current study has achieved satisfactory results among left-behind children in China, it remains unclear whether this conclusion can be extended to left-behind children with other cultural backgrounds, which should be tested in future studies.

## Conclusion

This study provides preliminary verification of the psychometric characteristics of the DASS-21 in Chinese left-behind children with the results of the ESEM model supporting the three-factor model. The DASS-21 was found to have good reliability, validity and measurement invariance among Chinese left-behind children, and can be used to assess their level of depression, anxiety and stress. This effort broadens the scope of application of the DASS-21, and has important practical significance for the study of the mental health of Chinese left-behind children.

## Data Availability

The datasets used and analysed during the current study available from the corresponding author on reasonable request.
